# BEATVIC, a body-oriented resilience training with elements of kickboxing for individuals with a psychotic disorder: study protocol of a multi-center RCT

**DOI:** 10.1186/s12888-016-0918-2

**Published:** 2016-07-08

**Authors:** Elisabeth C. D. van der Stouwe, Bertine de Vries, André Aleman, Johan Arends, Clement Waarheid, Aniek Meerdink, Erwin van der Helm, Jooske T. van Busschbach, Gerdina H. M. Pijnenborg

**Affiliations:** Department of Neuroscience, BCN Neuroimaging Center, University of Groningen, University Medical Center Groningen, Antonius Deusinglaan 2, 9713 AW Groningen, The Netherlands; University of Groningen, University Medical Center Groningen, University Center of Psychiatry, Rob Giel Onderzoekcentrum, Hanzeplein 1, 9713 GZ Groningen, The Netherlands; Department of Clinical Psychology, University of Groningen, Grote Kruisstraat 2/1, 9712 TS Groningen, The Netherlands; Department of Psychotic Disorders, GGZ-Drenthe, Dennenweg 9, 9404 LA Assen, The Netherlands; Helmsport, Vechtstraat 72B, 9725 CW Groningen, The Netherlands; Department of Movement and Education, Windesheim University of Applied Sciences, Campus 2-6, 8017 CA Zwolle, The Netherlands

**Keywords:** Psychotic disorder, Training, Assertiveness, Psychomotor, Nonverbal therapy, Kickboxing, Victimization, Social cognition, Self-esteem, Neuroimaging

## Abstract

**Background:**

Individuals with a psychotic disorder are at an increased risk of becoming victim of a crime or other forms of aggression. Research has revealed several possible risk factors (e.g. impaired social cognition, aggression regulation problems, assertiveness, self-stigma, self-esteem) for victimization in patients with a psychotic disorder. To address these risk factors and prevent victimization, we developed a body-oriented resilience training with elements of kickboxing: BEATVIC. The present study aims to evaluate the effectiveness of the intervention.

**Methods/Design:**

Seven mental health institutions in the Netherlands will participate in this study. Participants will be randomly assigned to either the BEATVIC training or the control condition: social activation. Follow-ups are at 6, 18 and 30 months. Short term effects on risk factors for victimization will be examined, since these are direct targets of the intervention and are thought to be mediators of victimization, the primary outcome of the intervention. The effect on victimization will be investigated at follow-up. In a subgroup of patients, fMRI scans will be made before and after the intervention period in order to assess potential neural changes associated with the effects of the training.

**Discussion:**

This study is the first to examine the effectiveness of an intervention targeted at victimization in psychosis. Methodological issues of the study are addressed in the discussion of this paper.

**Trial registration:**

Current Controlled Trials: ISRCTN21423535. Retrospectively registered 30-03-2016.

## Background

Contrary to popular belief, an individual with a psychotic disorder is more likely to become the victim of a crime or other forms of aggression, than to be the offender [1, 2]. Prevalence rates of victimization vary across studies from 20 % [3, 4] to 68 % [5], due to differences in study sample, in operationalization of victimization and in examined time frame (de Vries et al. in prep). In a recent study investigating victimization in people with a psychotic disorder, 39 % of patients had been severely physically threatened, 51 % of them reported physical violence and 32 % had been sexually victimized in the past year [3]. Moreover, a meta-analysis revealed that prevalence rates of victimization amongst severely mentally ill patients are 2 to 140 times higher than prevalence rates in the general population [6]. Again, this wide range of prevalence rates is due to methodological differences between studies. Most of the violence occurs in patients’ private environment, and is committed by people from their own social network [7]. Because victimization can have a major impact on the lives of already vulnerable patients, and ultimately entails high costs for society [1], a preventive intervention is needed.

In the past, several interventions to prevent victimization have been developed for severely mentally ill patients [8–11]. However, to date only one of these – a ‘street smart’ skills training for an urban setting – has been investigated by means of an observational pilot study [11] while the other interventions have not yet been empirically investigated, nor have they been implemented in clinical practice. Moreover, none of these interventions was developed for individuals with a psychotic disorder specifically [12], while the high victimization rates [1–3] and both psychotic patients and caregivers indicate the need for an intervention tailored to the needs of this specific group [13]. Therefore, we developed an intervention that aims to prevent victimization in people with psychotic disorders.

**Fig. 1:**
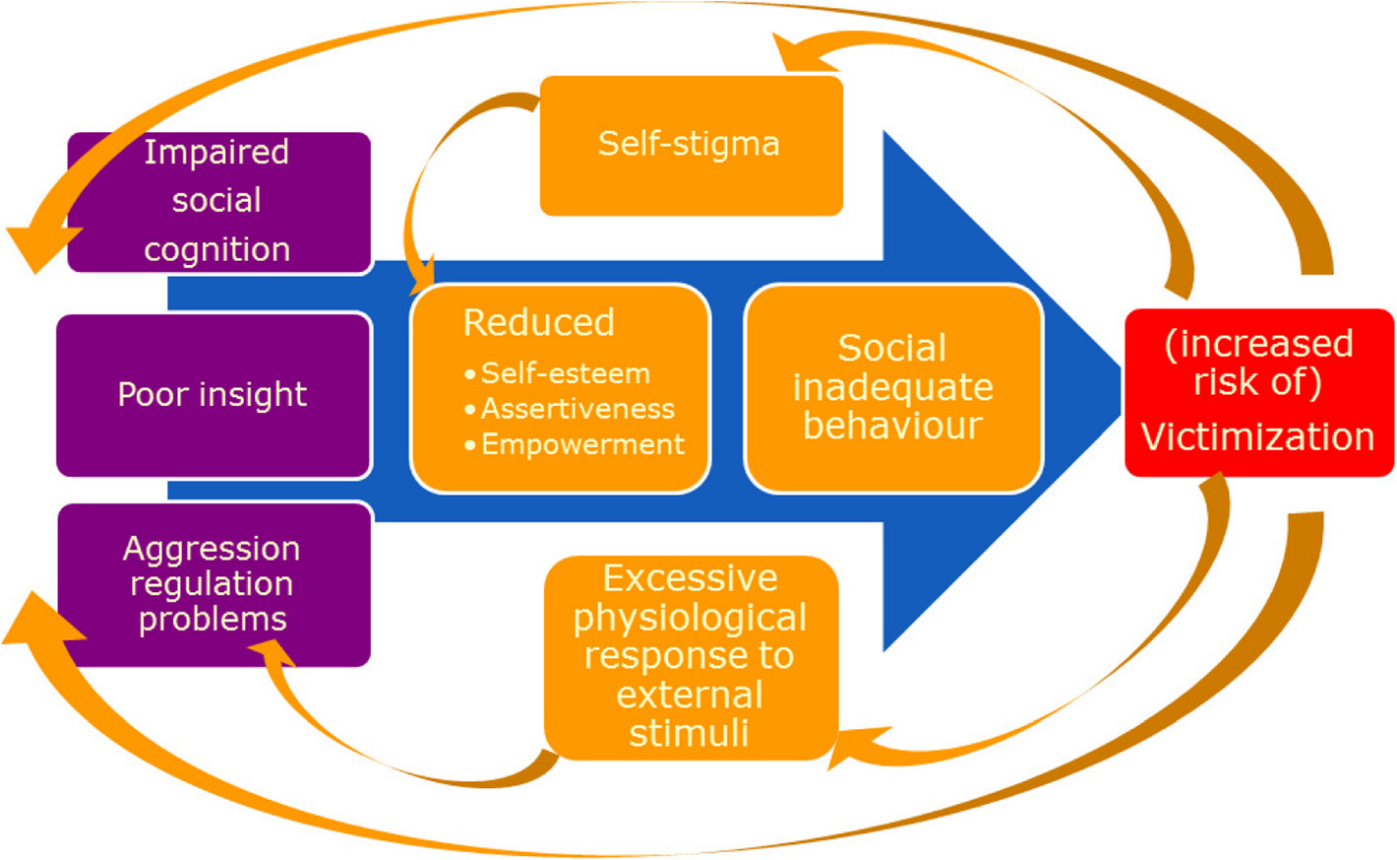
Victimization model

In order to design an effective intervention we selected targets that are potentially amendable to change by an intervention, and constructed a model (see Fig. 1) based on an analysis of risk factors as suggested by previous research. One of the potential risk factors might be deficits in social cognition [14]. Compared to individuals from the general population, people with psychotic disorders experience more difficulties recognizing facial expressions [15], body language [16], and emotional prosody [17]. This may lead to inadequate social behavior and - more specifically - may have a negative effect on their judgment of risky social situations. For instance, a patient may misinterpret the aggressive facial expression of another person, preventing him or her from leaving the setting before potential escalation. Another possible factor is insight. Poor insight in one’s own psychotic symptoms is one of the pathways to aggressive behavior [18] which may evoke aggressive behavior in others resulting in victimization [19]. Aggression regulation problems are associated with violent behavior in patients with a psychotic disorder and therefore these are another important risk factor [20]. Also, self-stigma may indirectly play a role in victimization; there are a lot of prejudice beliefs about mental illnesses and most patients are aware of these [21]. Self-stigma results in low self-efficacy [22], low self-esteem and reduced empowerment [23]. Consequently, individuals may be more prone to be victimized [24]. In turn, victimization may increase self-stigma and feelings of helplessness resulting in a vicious circle between self-stigma and victimization (Horsselenberg et al. in prep). Victimization not only affects self-stigma but also reinforces the other risk factors in the victimization model, increasing the chance of revictimization. For example, the traumatic experience of being a victim could lead to a stronger physiological response to external stressors [25] and a compromised inhibitory control [26]. People who experienced trauma or stress tend to react more aggressively in social situations [27, 28]. This aggressive response may elicit conflicts, again putting people at risk for victimization. Ultimately, patients can become trapped in a downward spiral.

We used this model to develop an intervention to decrease the risk of victimization in individuals with a psychotic disorder. We chose an experience based approach which combines body awareness exercises with physical activity, in contrast to primarily verbal interventions because victimization often occurs and derives from factors at a nonverbal level. This approach has its origin in what in some countries is called psychomotor therapy [29] or body-oriented psychotherapy [30]. Patients will be offered the opportunity to learn to recognize their own emotional and behavioral reactions to different social situations. In a safe and therapeutic environment, new behavior and for instance other body postures will be practiced stimulating adequate emotional awareness and emotion regulation. In addition, training with others and explicitly observing others during observational exercises facilitates participants to learn how others express themselves and to experiment within social interactions. These positive experiences resulting from the body-oriented approach will be mixed with less therapeutically embedded physical activities. Exercise therapies, varying from swimming to cardiovascular exercises, are known to have a positive effect on self-efficacy, self-esteem, social skills, and positive and negative symptoms in individuals with schizophrenia [31, 32]. All these effects show an overlap with the risk factors in the victimization model and could contribute to a decrease of victimization risk. The same holds for assertiveness training. Assertiveness training improves self-esteem, perceived control, assertiveness, and self-efficacy [33]. In the current training, assertive behavior such as setting boundaries can be practiced directly and indirectly, throughout all sessions. Finally, several studies suggest that martial arts could have a positive effect on aggression regulation and social interaction [34–36]. We chose kickboxing specifically because it enables participants to socially interact with each other and it requires continuous reading of each other’s body language and facial expression. Furthermore, kickboxing techniques are achievable for everyone regardless of someone’s physical condition which may result in experiences of success, enhancing self-esteem. To deal with all risk factors, a combination of mentioned approaches resulting in a body-oriented resilience training with elements of kickboxing was developed, from now on referred to as BEATVIC. The current paper presents the design of a multicenter randomized controlled trial which aims to evaluate the efficacy of the intervention.

### Research aims

Main aim of the study is to evaluate the effectiveness of BEATVIC. Primary outcome of this evaluation is victimization. It is expected that this effect will be mediated by risk factors of victimization (e.g. social cognition, internal stigma, assertiveness, self-esteem, aggression regulation, social behavior). Secondary outcome measures are negative symptoms, trauma, social participation, recovery and quality of life.

An additional aim of the study is to examine the effect of the training at the cerebral level, by means of functional Magnetic Resonance Imaging (fMRI). Particular emphasis will be on cerebral activation during social cognitive processes, since social cognition is a direct target of the intervention. We hypothesize enhanced involvement and connectivity of brain networks underlying social cognitive functioning in patients after the experimental intervention as compared to the control intervention.

## Methods/Design

The study is funded by the Netherlands Organization for Scientific Research (NWO grant nr 432-12-807). The study has been approved by the medical ethical board of University Medical Center Groningen, Groningen (number: NL52202.042.15). Trial number: ISRCTN21423535 (Current Controlled Trials).

### Design

The study is designed as a multi-center randomized controlled trial including a pretest, a posttest and three follow up assessments respectively at 6, 18 and 30 months. Patients allocated to the intervention group receive the BEATVIC training and patients allocated to the control group receive social activation sessions.

### Participants/Setting

A total of 120 patients will be included in the trial (see sample size calculation). Patients will be recruited in seven mental health institutions in the Netherlands.

Inclusion criteria are:

- □ A diagnosis in the psychotic spectrum, according to DSM-IV-TR criteria, verified by mini-SCAN.
- □ ≥ Eighteen years old.
- □ Ability to give informed consent.

Exclusion criteria are:

- □ Severe psychotic symptoms (mean positive symptoms > 5 measured by PANSS)
- □ Substance dependence (not substance abuse), verified by Miniscan.
- □ Co-morbid neurological disorder, verified by onsite therapist.
- □ Co-morbid personality disorder, verified by onsite therapist.
- □ Estimated IQ < 70, onsite therapist decides if the patients’ intelligence is sufficient for participation.
- □ Pregnancy.

A subsample of 44 patients will also participate in the fMRI part of the study. Additional exclusion criteria for the fMRI part of the study are: MR incompatible implants in the body, any risk of having metal particles in the eye (due to manual work without proper eye protections), tattoos containing red pigments, claustrophobia and refusal to be informed (by notifying the patients physician) of structural brain abnormalities that could be detected during the experiment.

### Sample size calculation

Sample size was computed using the IBM SPSS Sample Power program (Biostat, M. Borestein), http://www.power-analysis.com/about_biostat.htm. The effect size of our intervention is unknown. A common convention in this case is to set the effect size at 0.5, because a lower effect size would not be considered as clinically relevant. In order to find a medium effect size on our outcome measures with an alpha of 0.05 and a power of 0.80, a minimum of 48 participants per condition is required. Considering a drop-out of 25 %, we will include a total of 120 participants in our trial.

### Materials

A summary of the assessment/materials of the study is provided in Table 1.

**Table 1:** Overview of assessments

Instrument	Screening	Intake	Baseline	Post-assessment	Follow-up I	Follow-up II	Follow-up III
Questions for therapist	x						
MiniSCAN		x					
PANSS		x		x	x	x	x
Autobiographical questionnaire			x				
WOF			x	x			
Emotional faces paradigm			x	x			
MST			x	x			
Pedometer			x	x			
Faux Pas			x	x	x		
ZECV			x	x	x		
ISMI			x	x	x		
SIG			x	x	x		
SERS-SF			x	x	x		
PI			x	x	x		
IVM			x	x	x	x	x
CTS2			x	x	x	x	x
MANSA			x	x	x	x	x
NHS			x	x	x	x	x
SFS			x	x	x	x	x
BNSS			x	x	x	x	x
Screening risk of substance dependence			x	x	x	x	x

#### BEATVIC training protocol

BEATVIC consists of 20 weekly group training sessions of 75 min divided into five modules of four sessions each. Each session includes a warming-up, technical and thematic martial arts exercises, a cooling-down and a discussion of the addressed themes. All of the sessions will be led by a body-oriented therapist and an expert by experience, both trained by the team that developed the training protocol by means of a feasibility study. Throughout the modules, the amount of interpersonal contact as well as exercise intensity build up slowly in order to prevent tension or stress. At first, exercises will be performed individually or with a trainer, gradually participants will interact more and more with each other and exercise intensity will increase. In the introductory module, the trainers will create a safe group environment and participants and trainers will get familiar with one another. Basic kickboxing techniques and body posture will be taught and during exercises, special emphasis is on self-stigma, setting boundaries, awareness and respect for other’s boundaries and feeling tension. The second module ‘Recognizing dangerous behavior’ focuses on increasing social cognition and insight. During exercises, attention will be paid to how others move and react and participants will learn how to anticipate best themselves. Moreover, this modules aims to increase recognition of non-verbal communication, such as body postures, gestures and facial expressions that might lead to dangerous situations. The techniques used in this module are blocking and deflection of the kickboxing punches and kicks learned in the first module, which requires participants to read and adjust to each other’s body language. In the module ‘How others see me’, participants get more insight in underlying factors which affect their own behavior (e.g. emotions, characteristics of someone else) and how their behavior appears to others. Special emphasis is on automatic natural reactions to dangerous situations, such as fight, flight and fright. Participants will be made aware of typical offender- and victim patterns. Participants learn how to make use of posture, balance, voice and breath to feel and appear stronger. The exercises contain techniques which were taught in previous modules, but intensity will be enhanced gradually. In the fourth module ‘Coping with aggression’, participants will learn to detect and regulate one’s own aggression and they will learn to deal with aggressive behavior of others. The exercises focus on bodily signals of anger and tension and ways to reduce tension. Participants will practice with dosing and controlling their tension. By means of observational exercises, participants also learn to detect signals of tension in others. Furthermore, participants will get more insight in situations that usually evoke stress or tension. In the last module, trainers and participants can repeat exercises which were particular useful. Furthermore, trainers will stimulate participants to continue kickboxing outside of mental healthcare and thus stimulating social participation. For this purpose, a trial lesson at the local sports club will be organized for participants who are interested.

#### Control condition

The control condition consists of twenty weekly befriending meetings of 75 min. During these meetings, the main goal is to create a welcoming atmosphere in which participants can socially interact with each other in an informal setting. We developed a protocol which consists of four modules. During an introductory module participants and trainers will get familiar with one another by means of introductory games. The second module ‘Media’ consists of discussing the news, watching a documentary or tv show and watching and discussing online YouTube clips. In the following module ‘Hobbies’, participants get the opportunity to tell and show the group about their hobbies. Furthermore, participants can have a walk with each other, play music, read for themselves or color. In the last module, the group will play games and during the last session they will make a cake. Trainers will make sure participants talk about neutral topics, such as music, books, sports or the news. Befriending has often been used in studies investigating cognitive behavior therapy in the treatment for psychosis and seems to be a credible and acceptable control condition with regard to expectancy, enjoyment of therapy and drop-out rate [37]

#### Screening

On-site therapists will screen all patients in their caseload, based on the following questions:

- □ Is this patient diagnosed with a disorder in the psychosis spectrum?
- □ Does this patient currently have acute symptom characteristics of psychosis?
- □ Is this patient diagnosed with substance dependence?
- □ Is this patient diagnosed with a cluster B personality disorder?
- □ Is this patient diagnosed with a neurological disorder?
- □ Does this patient have an estimated IQ > 70?

#### Assessment

##### Behavioral measures

*Intake*

**Diagnosis** The *miniSCAN* [38] is a short version of the Schedule for Clinical Assessement in Neuropsychiatry (SCAN 2.1) and consists of structured interview questions regarding axis-I symptoms of the DSM-IV. In the current study, the miniSCAN will be used to verify a diagnosis in the psychosis spectrum and to verify that substance dependence is absent.

**Positive symptoms** The *Positive and Negative Symptom Scale* (PANSS) [39] is a semi-structured interview which contains 30 items divided into three subscales: positive symptoms, negative symptoms and general symptoms. Scores on positive items will be used to verify that florid psychosis (PANSS positive items > 5) is absent.

*Primary outcome measures*

**Victimization** The *Dutch Crime and Victimization Survey* (*Integrale veiligheidsmonitor)* [40] to a great extent resembles the International Crime Victimization Survey [41]. We will only use the subscale victimization, which contains questions regarding vandalism, threat and severe physical violence (Victrom) [42] as a measure for the amount of violent incidents over the different periods. The original five-year time-frame in the questions will be adapted at post-treatment assessments, in order to investigate victimization between two subsequent assessment points. The subscale safety perception which includes questions about safety in general and in one’s neighborhood will not serve as a primary outcome but as a possible explanatory variable. In addition to the IVM, we will use the revised *Conflict Tactics Scale* (CTS2) [43] as an extra indication of the frequency of violent incidents because it allows for the assessment of more subtle forms of victimization. The CTS2 is a widely used instrument in partner violence research and consist of 39 items. As in the *Dutch Crime and Victimization Survey*, the original five-year time-frame in the questions of the CTS2 will also be adapted at post-treatment assessments, in order to investigate victimization between two subsequent assessment points. Participants have to report to what extent the items apply to themselves or their partner in a given time period ranging on a scale from 1 ‘has never happened’ to 8 ‘more than twenty times’. Since we are interested in a broader range of social interactions we changed the questions from ‘partner’ to ‘someone’ as was done in an earlier epidemiological study on victimization in patients with SMI.

**Social cognition** The *Faux Pas task* [44] consists of ten stories, describing interpersonal, everyday situations. Some of these stories contain a ‘faux pas’: a person in the story does says something inappropriate due to a wrong interpretation of the others social signals. Participants have to detect these mistakes and infer how the character in the story at whom the faux pas is directed is feeling.

**Aggression regulation** The *Self-expression and Control Scale* (ZECV) [45] is a Dutch translation of 4 subscales of the State-Trait Anger Expression Inventory [46]. The questionnaire measures to what extent participants internalize or externalize feelings of anger and to what extent they can control that anger. The instrument consists of 40 items and participants respond by rating themselves on a scale ranging from 1 ‘almost never’ to 4 ‘almost always’. The ZECV has good to high psychometric properties.

**Internalized stigma** The *Internalized Stigma of Mental Illness Scale* (ISMI) [47] is designed to measure the subjective experience of stigma, and consists of subscales measuring Alienation, Stereotype Endorsement, Perceived Discrimination, Social withdrawal and Stigma Resistance. The questionnaire was developed in collaboration with people with mental illnesses and contains 29 items. The ISMI has a high internal consistency (α = .90) and test-retest reliability (*r* = .92).

**Social behavior** The *Interpersonal Behavior Scale* (*Schaal Interpersoonlijk Gedrag,* SIG) [48] measures social anxiety and social skills. For 50 items describing social situations, respondents have to rate the level of tension/discomfort they would feel, ranging from 1 ‘no discomfort’ to 5 ‘very much discomfort’, and the frequency of their occurrence in daily life.

**Self-esteem** The *Self-Esteem Rating Scale-Short Form* (SERS-SF) [49] is a 20-item self-report questionnaire which assesses self-esteem by means of a positive and negative self-esteem subscale. The instrument contains statements that are linked to social contacts, competency and achievement, and is validated for individuals with psychosis.

**Insight** The *Psychosis Insight Scale* (PI) [50] includes eight questions that address three dimensions of insight: awareness of illness, need for treatment and attribution of symptoms. The instrument is reliable, valid and sensitive to individual change.

*Secondary outcome measures*

**Quality of life** The *Manchester Short Assessment of Quality of Life* (MANSA) [51] is a short version of the Lancashire Quality of Life Profile (LQLP) [52], and has been developed to measure quality of life of psychiatric patients. The questionnaire consists of four objective items and twelve subjective items. The objective items assess victimization and accusation of crime in the past year, whether someone has a good friend and whether the participant had contact with this friend in the past week. The subjective items assess satisfaction with life as a whole, job, financial situation, leisure activities, accommodation, personal safety, sex life, people with whom the individual lives with, relationship with family, physical health, mental health and friendships on a seven-point rating scale (1 = negative extreme, 7- positive extreme). The internal consistency is sufficient (α = .74) to good (α = .81) [51].

**Recovery** The *National Recovery Scale* (*Nationale Herstelschaal,* NHS) [53] is a Dutch scale based on the Questionnaire about the Process of Recovery (QPR) [54]. The scale is developed to assess personal recovery of individuals with (severe) mental illnesses. The 26 item scale consists of two subscales: personal recovery and recovery with regard to interpersonal relations. The instrument is both reliable and valid.

**Societal participation** The *Social Functioning Scale* (SFS) [55] assesses social functioning and social participation. It consists of 78 items divided by seven subscales: social engagement/withdrawal, interpersonal communication, independence-competence, independence-performance, recreation, pro-social behavior and employment. The SFS is reliable, valid, sensitive and responsive to change.

**Symptoms** The *Brief Negative Symptom Scale* (BNSS) is an addition to the PANSS. This 13-item semi-structured interview has been developed to assess current level of negative symptoms into more detail. The instrument consists of 6 subscales: anhedonia, distress, asociality, avolition, blunted affect and alogia.

**Trauma** The *Trauma Screening Questionnaire* (TSQ) [56] examines whether participants suffer from trauma and to what extent participants cope with trauma. The TSQ is a short screening instrument which consists of five re-experiencing items and five arousal items derived from the DMS-IV PTSD criteria. The sensitivity and specificity of the TSQ are high [57].

**Physical activation** Participants will wear a validated and reliable pedometer (Yamax) [58] for the continuous recording of physical activity during a period of 7 days. The Yamax EX 510 is a light and small device which can be worn in a pocket or in a handbag. Pedometers are a valuable tool for motion analysis in clinical populations [58].

**Endurance** The Modified Shuttle Test is a sub-maximal test to measure endurance. Participants have to walk between two points. A beep indicates when the participant has to be at the next point. The interval between the beeps becomes shorter every level. The outcome measure is the amount of meters a participant is able to walk or run between the two points. The MST is shown to give reliable test results and is appropriate to perform for people who suffer from somatic disorders or decreased fitness [59].

*Covariates*

**Substance use** The *Screening risk of substance dependence* (*Screening risico op verslavingsproblemen)* [60] is a self-report questionnaire which consists of eleven questions regarding how much alcohol and drugs the participant uses in one week or month.

**Biographical characteristics** The autobiographical questionnaire is a form which contains questions about gender, family contact, age, medication use and residential area.

##### fMRI

**Social cognition** The *Wall of faces task* (WOF) we applied was based on a version in a previous study [61] in which it was used to probe neural processes underlying affective appraisal of various simultaneously presented faces. Each trial, 32 faces will be presented which vary in angry/happy (experimental condition) and male/female ratio (control condition). The task consists of ambiguous (16 of each face type) and unambiguous (6/26) trials. Participants will be asked to identify the predominant emotion (experimental condition) or the predominant gender (control condition). During face presentation and response time, the condition “Angry - Happy” or “Female - Male” will displayed on the screen. Each trial, the faces will be presented for three seconds, followed by a response time of 1,5 s.

Faces will be obtained from the Karolinska Directed Emotional Faces database [62]. The task allows for investigation of brain activation associated with social cognitive processes.

**Threat-response** In the emotional faces paradigm, participants will complete a gender discrimination task including 16 blocks of individual neutral faces, 16 blocks of happy faces, 16 blocks of fearful faces and 16 blocks of angry faces. Each block contains six trials, including three to five face trials and one to three null trials consisting of a fixation cross. Within blocks, face trials and null trials will be mingled at random. Face trials comprise the stimulus presented for 600 ms and an interstimulus interval of 500 ms during which a fixation cross will be presented. Null trials consist of a fixation cross presented for 1100 ms. Faces will be obtained from the Karolinska Directed Emotional Faces database [62]. The task takes 9 min and allows for examination of basal threat-related brain response.

#### Procedure

Onsite therapists will screen patients based on selection questions regarding in- and exclusion criteria. Patients who meet the criteria according to the therapists, will be contacted and asked if they are interested in participating in the study. Interested patients will be provided with an information letter. Following, patients have a two-week period to consider final participation. If a patient is willing to participate, an intake is scheduled to confirm that the patient meets the inclusion criteria, by means of the MiniSCAN and the PANSS and if applicable, an MRI-checklist and a written informed consent will be obtained. Patients who are eligible for participation will be randomly allocated to either the intervention condition or the control condition. Randomization will be done for each center separately to guarantee a comparable number of participants in both groups, when the required number of patients (20) is included, or when the first participant was included more than 6 weeks ago while > 12 participants are included. This will be performed by an independent team of researchers which is not involved in the trial. Randomization will be stratified by gender and participation in the fMRI substudy, such that participants have a 50 % chance to be allocated to the intervention condition. The trainers of BEATVIC will receive a train-the-trainer course that consists of four sessions of 2,5 h during which most important exercises are trained and the most important background is discussed. When the BEATVIC training has started, the study investigators and body-oriented therapist of the training team will monthly visit a session in order to monitor the training and to supervise the onsite trainers. Similarly, the organizers of the befriending sessions will be trained by the study investigators and will be supervised monthly. At all sites, trained interviewers are available who will be blinded to the study condition. Trainers and patients cannot be blinded after treatment allotment. Patients will be instructed not to inform the assessor about the study condition they were allocated to. Assessors will be asked to report whether they had an idea to which study group the assessed patient was been allocated. If this is the case and this is correct, the interview will be marked. By means of a sensitivity analysis it will be checked whether results are affected after including marked data and whether there should be controlled for expectation of the assessors. Assessment takes place before (T1), directly after (T2), six months after (T3), 18 months after (T4), and 30 months after the training (T5) at the particular site. All fMRI scans take place before (T1) and directly after (T2) treatment at the Neuroimaging Center of the UMCG in Groningen.

## Statistical analysis

### Behavioral data

Analysis will be performed according to the *intention to treat principle* [63]. Differences in scores between pretest, posttest and follow-up assessments and between condition groups will be examined for each of the dependent variables. This requires the use of multi-level modeling procedures [64], with assessment time (pretest, posttest, follow-up I/II/II) at level 1, participants at level 2 and condition as independent variable. Age, substance use, medication and gender will be added as covariates to the models in case there are significant differences between the intervention group and the control group. For each of the dependent variables, the primary outcomes and the risk factors, a multilevel model will be constructed using the program MlwiN. To all models, dummy variables for both levels and interactions between dummy variables will be added as fixed factors. Statistical significance of the regression effects will be tested using the *T*-test. In all analyses, a *p*-value < .05 will be considered statistically significant.

### fMRI data

Neuroimaging data will be preprocessed using statistical parametric mapping 8 (SPM 8; Wellcome Department of Cognitive Neurology London, UK; http://www.fil.ion.ucl.ac.uk) in Matlab version 7.8.0 (Mathworks, Natick USA). Functional images will be corrected for slice timing and will be realigned to correct for head motion. Next, images will be coregistered to the anatomical scan of the participant. Coregistrations will be controlled manually for each participant. Following, all functional scans will be normalized to MNI space and then spatially smoothed with a 8 mm full-width half-maximum (FWHM) isotropic Gaussian Kernal. Preprocessed data will be analyzed using traditional General Linear Model (GLM) voxel-wise analyses. We will analyze the fMRI data according to the ROI-based method used by Subramanian et al. (2014) [65], based on Poldrack (2007) [66]. Baseline victimization scores as measured by the IVM will be entered as regressors. Moreover, the effects of the training will be evaluated via 2 (Group: intervention, control) × 2 (Assessment: pre-assessment, post-assessment) interaction tests. Furthermore, we will study connectivity during a resting state fMRI to investigate changes in intrinsic networks.

## Discussion

Individuals with a psychotic disorder are at an increased risk of becoming victim of a crime or other forms of aggression [67]. This trial will be the first to evaluate the effectiveness of a preventive intervention targeted at victimization for people with psychotic disorders. Effects will be examined at both a behavioral level by means of interviews and questionnaires and a cerebral level with fMRI scans. If proven effective, BEATVIC can be implemented in mental health care and contribute to the safety and well-being of psychotic patients. Especially, since therapists are currently left empty-handed when it comes to prevention of victimization. Furthermore, this study may improve insight in victimization and its risk factors. The longitudinal design enables the investigation of the role of risk factors on victimization in the long term. Moreover, the design allows to investigate whether improvement of risk factors results in a decrease of incidents.

A methodological difficulty of this study, and of study designs with follow-up assessments in general, is the drop-out risk. We will control for drop-out by the inclusion of 25 % extra participants to ensure statistical power will be maintained. Another more general drawback involves the use of self-report measures. Common criticism on self-report questionnaires concerns the fact that they require insight in one’s behavior, they may be subject of social desirability bias or biases related to timing. However, measures that will be used have been proven reliable and valid. In addition, the current study also includes fMRI tasks, interviews, a neuropsychological test and physical measures. An issue more specific to this study concerns the selection of patients. Because of the exclusion of patients with acute psychotic symptoms characteristics of psychosis and patients with co-morbid personality disorders, the sample may not include the most severely ill patients. Despite the fact that trauma or post traumatic stress disorder (PTSD) has long been a reason for caution with regard to the treatment of psychosis, this is not an exclusion criterion. Recent studies showed that it is safe and effective to treat psychosis and co-morbid PTSD [68]. Moreover, body- and movement-oriented interventions are common for traumatized individuals [69, 70]. Finally, vulnerable patients often live in an isolated environment which protects them from potential harm. The training motivates patients to take initiative and get out of their isolation which might heighten the risk of victimization. In an unpublished feasibility study prior to this trial, participants appeared to be more empowered, took more initiative and got more self-confident at the end of the training. After the study completion, a subgroup of participants decided to attend kickboxing lessons in a regular gym under supervision of the expert by experience. Corroboration of such results in larger, randomized controlled trials may warrant inclusion of this approach in regular practice.
